# Reliability of plasma HIV viral load testing beyond 24 hours: Insights gained from a study in a routine diagnostic laboratory

**DOI:** 10.1371/journal.pone.0219381

**Published:** 2019-07-03

**Authors:** Diana Hardie, Stephen Korsman, Sharifa Ameer, Lara Vojnov, Nei-Yuan Hsiao

**Affiliations:** 1 Division of Virology, Department of Pathology, University of Cape Town, Cape Town, South Africa; 2 National Health Laboratory Service, Cape Town, South Africa; 3 World Health Organization, Geneva, Switzerland; "INSERM", FRANCE

## Abstract

**Background:**

Viral load testing is key to monitoring response to anti-retroviral therapy (ART). However, in lower and middle income countries with large epidemics, pre-analytical challenges threaten the quality of testing. It is unknown how much delayed processing and adverse storage affects the validity of results. The aim of this study was to determine the impact of delayed testing and warmer storage conditions on HIV RNA stability in diagnostic samples.

**Methods:**

1194 samples, collected in EDTA or plasma preparation (PPT) tubes, were studied. Immediately after initial testing, primary tubes were stored for 72, 96 or 168 hours at 4°C, 20°C or 30°C. The viral load was then repeated and the 2 results were compared.

**Results:**

Viral loads were very stable, with <0.5 log copies/ml median difference noted between paired tests for all storage times and temperatures. The viral load in samples stored for up to a week reliably differentiated between ART-suppressed and failing patients in 98.83% of instances. However, re-centrifugation immediately prior to repeat testing was essential to avoid falsely elevated readings, probably due to contamination of plasma with cell-associated viral nucleic acids. Approximately 20% of samples with initially undetectable viral loads were weakly positive (<100 copies/mL) on repeat. This was not exacerbated by duration or temperature of storage.

**Conclusion:**

Viral RNA in diagnostic samples is stable well beyond currently recommended limits. However, when testing stored primary samples, contamination of plasma with cellular material easily occurs. Low viral loads (<100copies/mL) in samples stored in this way should be interpreted with caution.

## Introduction

Viral load (VL) testing is a key component of achieving and monitoring progress towards the “third 90” of the UNAIDS 90:90:90 targets. South Africa (SA) has made great progress in improving access to testing and has the world’s largest monitoring program. Close to 5 million viral loads were performed by public laboratories in SA in 2017 [1]. However, logistical and pre-analytical challenges such as transporting, registering, centrifuging and storing samples threaten the quality of the testing service.

Currently manufacturers of high throughput VL platforms recommend that whole blood samples should be centrifuged within 6 to 24 hours of collection and plasma should either be frozen at—80°C or tested within 24 hours of sample collection [2] [3]. In resource-limited settings, this is often not achievable. Samples may reach the laboratory more than 24 hours after collection and are generally stored at 4°C in their primary tubes until testing. The extent to which these pre-analytical factors compromise accuracy of results is unknown. Past laboratory simulation studies [4] [5] and a systematic review [6] that performed a meta-analysis of these laboratory studies, have suggested that viral RNA decay may be slower than previously thought and that testing can be performed for some time beyond the recommended 6 to 24 hour cut off without compromising accuracy.

Two types of sample tubes are used in South Africa, namely standard ethylene diamine tetra acetic acid (EDTA) and plasma preparation (PPT) tubes. Use of the latter was introduced to improve RNA stability in samples where manufacturer timelines are not achievable and were locally validated [7]. PPT tubes contain an inert gel and spray-dried EDTA. During centrifugation, the gel migrates and forms a barrier between the plasma and cellular elements. However, there have been concerns that measurements in PPT tubes may be affected by pre-analytical processing [8] and [9]. In particular, there are reports that RNA levels may be higher than in standard EDTA tubes [10]. Also, adequate separation of cellular elements may not always occur and this can result in falsely elevated viral load readings if testing is delayed [11]. For this reason it is routine practice to re-centrifuge samples just prior to viral load testing, at public sector laboratories in South Africa.

The aim of this study was to assess stability of HIV RNA in routine diagnostic samples stored in their primary tubes (both EDTA and PPT) and to determine to what extent measurements are affected by sub-optimal handling and storage.

### Study objectives

1. To determine the impact of delay and storage temperature on the reproducibility of VL measurements in HIV RNA positive samples
2. To determine the impact of delay and storage temperature on the reproducibility of VL measurements in samples with undetectable HIV RNA.
3. To assess the value of repeat centrifugation immediately prior to testing when VL testing is delayed.

## Methods

### The effect of duration and temperature of storage on viral load values in PPT and EDTA tubes

Routine diagnostic specimens collected from patients on ART as part of the South African national antiretroviral programme in and around Cape Town were studied. Samples were processed according to the local standard operating procedure, briefly outlined as follows: On arrival at the laboratory, patient and sample information, including date and time of collection are captured on the laboratory information system and samples are centrifuged for 10 minutes at relative centrifugal force (RCF) 3,273g. They are then transferred to the bench where HIV VL testing on primary tube EDTA or PPT samples is performed on the Roche Cobas AmpliPrep, Cobas TaqMan (CAP/CTM) version 2.0 assay. Using a 1 mL input, this assay has a dynamic range from 20 to 10 000 000 copies/mL (1.3–7 log_10_). The delay between sample collection and initial VL testing is recorded as time to test 1. The result of test 1 was reported as the final result for the purpose of patient management. Thereafter, the samples were de-identified and assigned a separate study number. Further testing was not part of routine laboratory process.

Immediately after plasma was aspirated from the primary tube for test one (and the testing process began), the primary tubes were visually inspected. Those with sufficient (>1 mL) residual plasma were selected and stored at 4°C, 20°C or 30°C for a pre-determined interval of 72, 96 or 168 hours. Approximately 30% of PPT and 20% of EDTA samples had enough residual plasma for a second test. After storage at the specified temperature and time, primary tubes were re-centrifuged (3,273g for 10 minutes), 1mL of residual plasma was manually pipetted from the tube and the VL was repeated. The second VL result was captured as test 2. A figure illustrating the study plan is included in supplementary digital content, **S1 Fig**.

### Impact of re-centrifugation immediately prior to viral load testing

In a second set of experiments, routine diagnostic samples were processed as before, but re-centrifugation prior to the second test was omitted. These samples were also subjected to delayed testing with different storage conditions as above so the impact of repeat centrifugation was separately assessed. 37 EDTA samples (32 of which were undetectable in test 1) and 46 PPT samples (of which 29 were undetectable) were tested.

### Analysis

Data were analysed using Stata Version 12.0 (Stata Corporation, College Station, Texas, USA). For analysis, samples with an undetectable VL (LDL, or lower than detectable limits) on test 1 or test 2, were assigned a value of 1 copy/mL. Samples with a detectable VL, but less than 20 copies/mL, were assigned a value of 19 copies/mL. Samples with a VL >20 copies/mL were assigned the value reported by the analyser. The impact of delayed testing, storage temperature and repeat centrifugation were assessed in three separate analyses. First, the VL change among samples with a detectable test 1 VL (>20 copies/ml) were assessed. The proportion of instances where the test 2 VL was within 0.5 log_10_ copies/mL of test 1 for each storage temperature and time category was determined. This cut off was selected because in clinical practice, only VL changes >0.5log copies per ml are considered clinically significant [12]. The primary stability estimate was the median change in VL after the 72, 96 and 168 hour intervals and variability in this change was expressed in interquartile ranges. In addition, the proportion of instances where the test 2 VL result was within 0.5 log10 copies/mL of test 1 for each storage temperature and time category was determined. Results of samples collected in PPT tubes and EDTA tubes were analysed separately. Second, samples with undetectable RNA in test 1 were assessed to determine reproducibility of measurement. Finally, repeat VL measurements in an additional set of samples that was not re-centrifuged prior to retest was analysed separately.

Ethics approval for the study was obtained from the University of Cape Town Human Research Ethics Committee, reference number HREC 159/2019.

## Results

### Samples

For the main study, 1194 routine diagnostic samples, including 608 samples collected in EDTA and 586 in PPT tubes were studied. 441 (36.9%) of samples had undetectable VL on first test. in the remaining 753 samples, the viral load in test 1 was detectable and ranged between 19 –>1,000,000 copies/mL. A breakdown of the first test VL measurements is given in **Table 1**.

**Table 1:** Breakdown of study sample characteristics.

Sample Type:	EDTA	PPT	Total
Number:	608	586	1,194
1st viral load category (copies/mL):			
Undetectable (LDL)	246	195	441
<100	170	84	254
2-3LOG	64	97	161
3-4LOG	42	76	118
4-5LOG	54	85	139
>5LOG	32	49	81
Time to first viral load:			
<24hr	357 (53.68%)	308 (46.32%)	665
≥24hr	251 (47.45%)	278 (52.55%)	529
Samples in each delayed experiment category:			
Retest 72hr:	207	213	420
4°C	68	75	
20°C	87	82	
30°C	52	56	
Retest 96hr:	195	196	391
4°C	76	81	
20°C	75	57	
30°C	44	58	
Retest 168hr:	206	177	383
4°C	65	57	
20°C	86	72	
30°C	55	48	

In 665 (55%) samples the first VL was performed within 24 hours of sample collection. For the remaining 529 samples, the first test was performed between 1–7 days of sample collection. Delayed testing mostly occurred in samples referred from distant laboratories, or due to technical problems with instrumentation in the laboratory.

In the experiment where repeat centrifugation was omitted prior to repeat VL testing, 83 samples (37 EDTA and 46 PPT) were tested.

### The effect of delayed testing on initially RNA positive samples stored at 4°, 20° and 30°C

**Fig 1:**
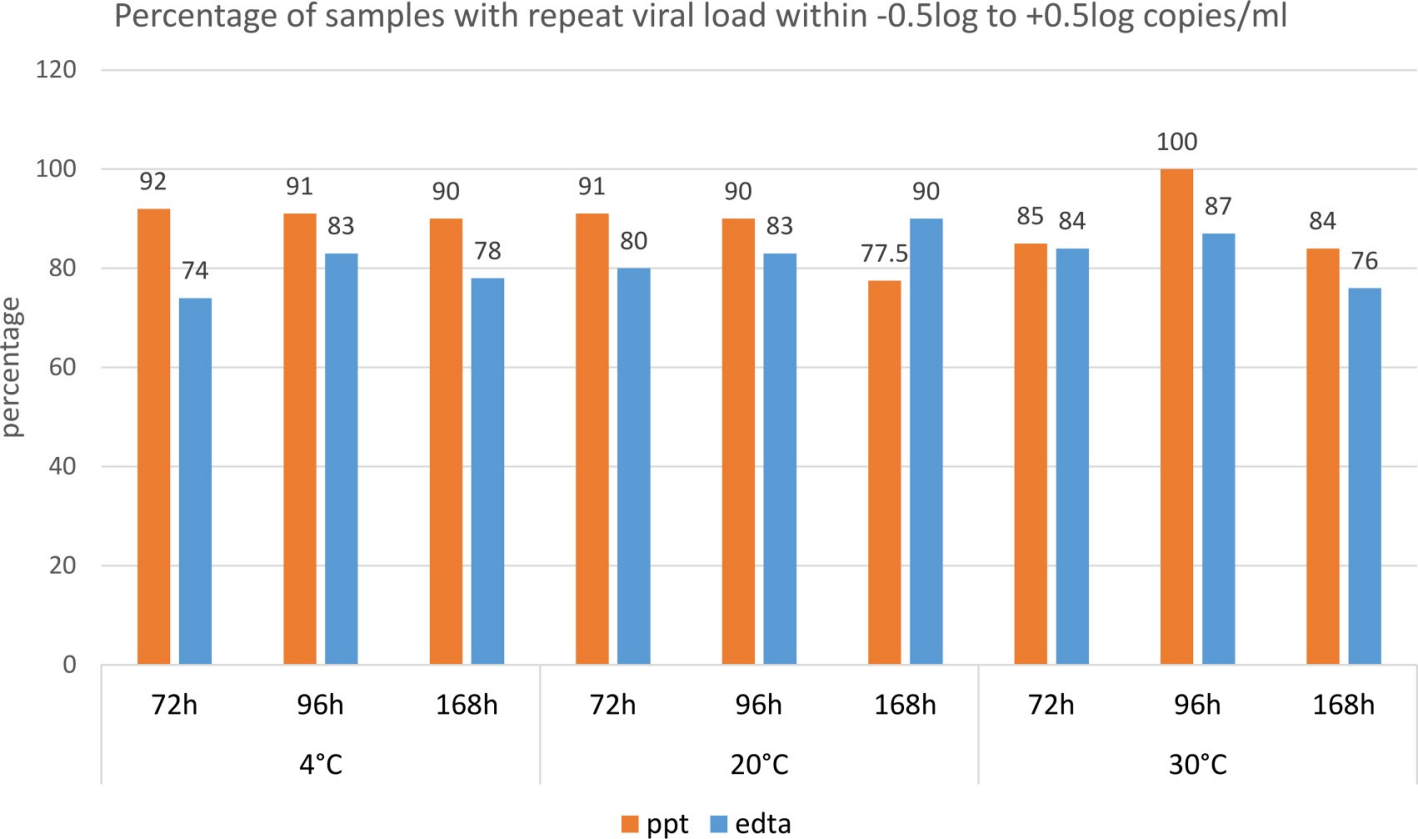
The proportion of samples in EDTA or PPT tubes with repeat viral load within -0.5 to +0.5 log copies per mL of the first test for each storage category. In 78–100% of PPT samples and 74–90% of EDTA samples the repeat viral load was within +/- 0.5 log copies/mL.

Overall, 86.6% (95%CI 83.7–89.2%) of all test 2 VL results were within 0.5 log copies/mL of test 1. This VL concordance rate was similar, at 84.0% (95%CI 79.7–87.7%) for the subset of samples where the first VL test had been performed within 24 hours of sample collection (within the current recommended optimal testing interval). As no significant differences were detected between the 2 data sets, only the results of the complete data set are reported here. A viral load concordance rate of 90.3% (95%CI 86.9–93.1%) was observed for the PPT samples overall, whereas the 2 VL readings were within 0.5 log copies/mL in 81.4% (95% CI 76.1–85.9%) for EDTA samples. There was no rising trend in the proportion of samples with a viral load difference of >0.5log copies/mL between tests 1 and 2, with increasing time or temperature of storage. (**Fig 1**) Variability was slightly greater in samples in EDTA tubes, but otherwise trends were similar (as determined by median and IQR of log viral load change in the different time/temperature storage categories).

**Fig 2:**
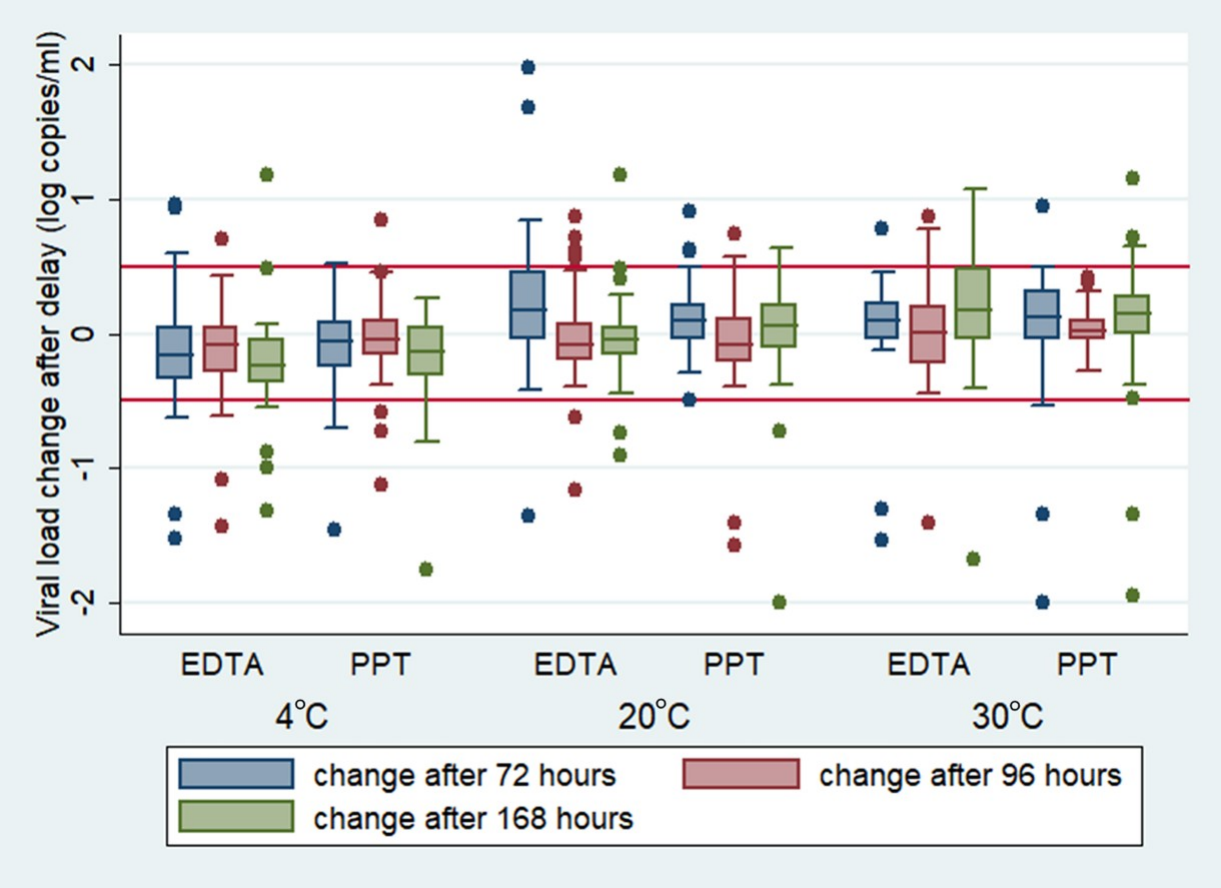
Box and whisker plots of log viral load change (test 2-test 1) in EDTA and PPT samples at 4°C, 20°C and 30°C: All samples with detectable viral load >19 copies per ml in test 1 were analysed. **Median and IQR for each temperature/storage category is given.** Plus and minus 0.5log viral load change indicated by solid red lines.

Fig 2 illustrates changes in log VL at each storage temperature and time point. For samples stored at 4°C, a median VL decrease was consistent for both sample types (EDTA and PPT) and over time, but the net effect was small, with all the interquartile ranges well within 0.5 log copies/ml compared to the initial VL results. At 20°C the trend was less apparent with some testing intervals resulting in slight increase, while others remained similar to original testing. Once again the VL changes were not significant, as none of the interquartile ranges cross the 0.5log difference line. In samples stored at 30°C, median values were mostly higher than baseline values. (Median log copies/ml differences for each sample category are given in supplementary digital content, S1 Table.) Greater variability was observed in samples collected in EDTA tubes, in particular the 72 hour samples at 20°C and 168 hour samples at 30°C.

WHO guidelines for management of patients on ART advise a level of >1000 copies/mL as the threshold indicative of treatment failure [13]. Thus the study results were analysed according to whether the difference between tests 1 and 2 was great enough to result in misclassification of a patient’s status (failure vs suppression). Of 1194 samples tested, a potential misclassification may have occurred in 14 (8 EDTA and 6 PPT) instances, or 1.17% samples. The discordant results are listed in supplemental digital content, **S2 Table.** In 10 the initial VL (range 1–890 copies/ml) increased to between 1000–70086 copies/mL and in 4 samples the level (range 1674–2940 copies/ml) decreased on repeat to between 500–846 copies/mL.

### The effect of delayed testing on samples with undetectable HIV RNA in test 1, stored at 4°, 20° and 30°C

**Fig 3:**
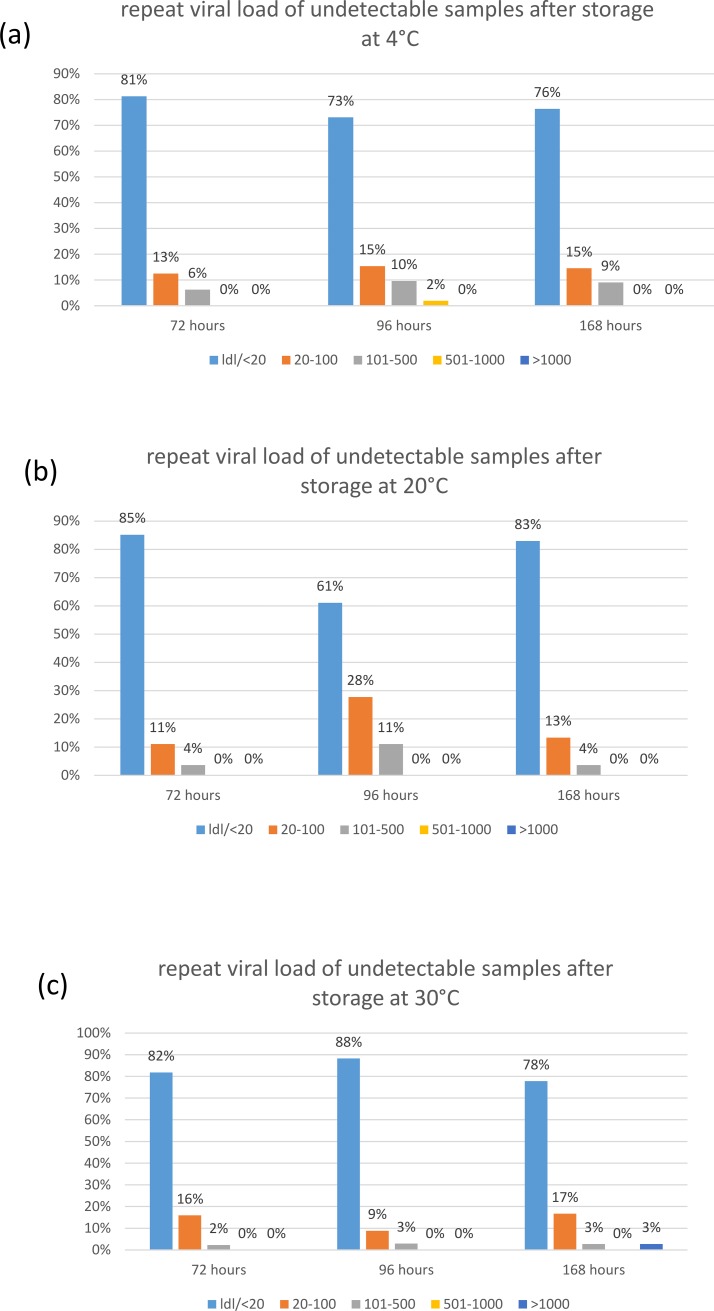
Repeat viral load results of samples that were undetectable in test 1: The proportion of samples that were undetectable or <20 copies/mL, 20–100 copies/mL, 100–500 copies/mL, 500–1000 copies/mL or >1000 copies/mL on repeat testing are shown. In (a) samples that were stored at 4°C for 72, 96 and 168 hours, (b) samples that were stored at 20°C and (c) samples that were stored at 30°C.

The stability of samples that were RNA negative at test one was also tested. 441 samples (195 PPT and 246 EDTA) had undetectable RNA at test one. On retesting after storage, the VL in 79.1% of samples remained undetectable or <20 copies/mL. In the 20.9% of samples that had a detectable value on the 2^nd^ test, RNA levels were mostly low. Overall, in 14.7% of samples the second viral load was <100 copies/mL, in 5.7% it was 101–500 copies/mL and in 0.4% > 500 copies/mL. Though no statistical test was used to confirm this, the proportion of samples that had a detectable viral load on 2^nd^ test did not appear to be affected by storage time, temperature or type of collection tube. **Fig 3(A)** and **3(B)**.

### Re-centrifugation of stored samples prior to repeat testing is essential to ensure accurate viral load readings:

**Fig 4:**
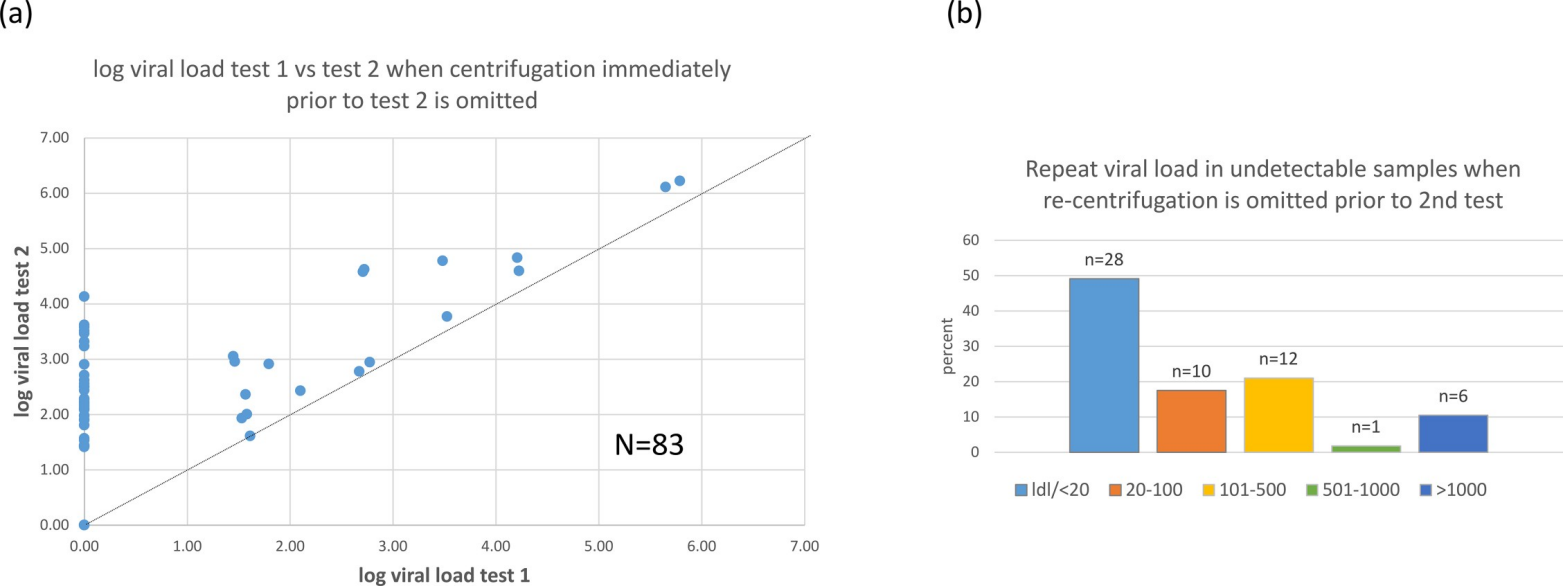
Omitting centrifugation immediately prior to repeat testing is associated with a higher viral load in the second test: In (a), the log value for each sample in test 1 (X-axis) is plotted against the log value in test 2 (Y-axis). Line indicates an ideal regression line, r^2^ = 1. In (b) the repeat results of samples that were undetectable (LDL) in test 1 are graphed according to the following categories: undetectable or <20 copies/mL, 20–100 copies/mL, 101–500 copies/mL, 501–1000 copies/mL or >1000 copies/mL.

When repeat centrifugation was omitted just prior to the 2^nd^ test, the RNA level in test 2 increased substantially in some samples. (**Fig 4(A))** This was observed in both EDTA and PPT samples and was evident by 48 hours after (prior) centrifugation. The VL remained within 0.5 log copies/mL in test 2 in only 41 of the 83 (49%) samples tested in this experiment. Of note 29 of 57 samples with initially undetectable HIV RNA had detectable RNA in the 2^nd^ test: 10 (17.5%) were < 100 copies/ml, 12 (21%) were 101–500 copies/ml, 1 (1.8%) was 501–1000 copies/ml and 6 (10.5%) were >1000 copies/ml. (**Fig 4(B)**) Overall, the median increase was 0.63 log copies/mL (range 0.00–4.13) in test 2.

## Discussion

Viral load test manufacturers advise that plasma separation should be performed within 24 hours of sample collection to ensure a reliable test result. In large ARV programmes such as that in South Africa where VL testing is centralised, a significant proportion of samples from rural areas reach laboratories beyond this recommended time interval and it is unknown to what extent a delay in testing compromises results. The fact that, in this study only 56% of samples had been tested within 24 hours of sample collection demonstrates this very challenge. For this reason, research is ongoing into the use of alternative sample types, such as dried blood spots (DBS) or plasma separation cards. However, these approaches have drawbacks: only small amounts of blood can be collected which reduces assay sensitivity, and with DBS, the sample is contaminated with viral nucleic acids from cellular elements, which makes results difficult to interpret [14]. Also, the practicality of performing VL testing on a variety of different sample types is questionable. This could create challenges in workflow and ultimately prolong turnaround times in a busy VL laboratory. Testing of liquid plasma remains the sample of choice. Thus there is an urgent need to determine for how long viral RNA is stable after collection. In this study we set out to investigate this in routine diagnostic samples. 1194 clinical samples with a range of initial RNA levels, from undetectable to >6 log copies/mL were studied.

Factors that could alter viral RNA levels or affect the accuracy of measurement when testing is delayed include release of intra-cellular viral nucleic acids [9] [11], ineffective removal of platelets during centrifugation [15] [16], decay of plasma viral RNA [4] [5] or haemolysis and release of other PCR inhibitors from the specimen. In this study we found that HIV RNA in samples stored in their primary tubes was unexpectedly stable. Even after prolonged storage at 30°C, the variation between test 1 and 2 in clinical samples was on average well within acceptable variation [12]. In the vast majority of samples, RNA levels were within 0.5 log copies/mL of each other. These findings support earlier studies which suggest that HIV RNA could be more stable in clinical samples than is implied by the strict timelines indicated in manufacturer’s kit instructions [2][3]. These findings suggest that intact virus particles in plasma are quite stable, as viral RNA remains protected by its capsid and nucleoproteins. EDTA added to the tubes may well further contribute to RNA stability. Its mode of action is to chelate calcium and other metal ions and this inhibits many cellular enzymes which might otherwise have degraded virus particles and free RNA [17] [18]. It is for this reason that EDTA is preferred to other anticoagulants for molecular testing in blood.

Viral RNA appeared to decay in samples stored at 4°C, less so at room temperature and actually increased at 30°C, though not significantly. This suggests that the VL is impacted by a number of opposing factors and the measured result is the sum of these. Two identifiable factors are RNA decay of free plasma virus and release of intra-cellular viral nucleic acids. If these factors are indeed the dominant processes, it follows that storage at 4°C slows down release of intracellular RNA (or virus) from cells. Hence, the dominant trend at this temperature is due to decay of free virus. However, it must be noted that the median change was small, only -0.24 log copies/mL after 168 hours for EDTA and -0.13 log copies/mL for PPT samples. At room temperature, both processes partially cancel each other out, hence the net effect is an even smaller difference. At 30°C, the dominant effect appears to be viral RNA release into the plasma. However, even at this temperature, the change was within acceptable limits. In 1.17% of instances (14 samples) the difference between tests 1 and 2 were sufficient to result in a change in the patient’s status, based on WHO guidelines [13]. Eight of these were EDTA samples and in most instances, the 2^nd^ viral load was higher. Variability was consistently less in samples collected in PPT tubes. This is likely to be due to lower (but not negligible) exposure to cellular elements in PPT tubes during extended storage.

A major factor that impacted on reproducibility was omitting the repeat centrifugation step just before the second test. This affected a significant proportion of samples in both PPT and EDTA tubes in this separate experiment. The repeat VL reading could be higher by up to 4.13 log copies/mL. This suggests that cellular elements containing viral nucleic acids, or inadequately removed platelets with trapped HIV particles, diffused into the liquid phase during storage and were aspirated during the second test. Re-centrifugation immediately prior to testing was largely effective at preventing this. In PPT tubes the gel should prevent aspiration of cellular elements during testing. However, inspection of these tubes often revealed small amounts of cellular material at the gel-fluid interface, indicating that effective separation of plasma from cellular elements does not always occur completely. This phenomenon has been described [10] and it is routine practice at viral load testing laboratories in South Africa to re-centrifuge samples just prior to testing, even if samples have been centrifuged previously.

Patients on ARVs have variable amounts of intra-cellular viral RNA and DNA (both are detectable with viral load assays if cellular material is aspirated during testing) [14]. While HIV RNA decay in plasma is rapid after initiation of effective ART, decay of intracellular proviral and episomal DNA is slower and ongoing viral mRNA continues to be synthesised despite therapy [19]; [20]. Thus viral nucleic acids continue to be detectable in cellular compartments long after initiation of ART. While most samples that were undetectable at test 1 remained undetectable at test 2, 20.9% of samples had a low level of RNA detectable on test 2 (with a VL mostly <2log copies/mL). The proportion of samples that became positive was similar in all delayed testing groups which suggests that delay in testing was not the main cause of this phenomenon. The finding can partly be explained by the recognised variability in VL detection when RNA levels are close to the limit of detection of the assay. But it could also be due to inadvertent aspiration of cellular elements, including platelets. Indeed, the study design could have increased the chances of detecting cell associated viral nucleic acids in the second test. Due to a lower volume of plasma available for the repeat test, the risk of aspirating cellular material would have been greater. However, as low volume samples are a frequent occurrence in our setting, it is important to recognise this potential problem. Thus low levels of viral RNA detected in samples stored and tested in this way should be interpreted with caution. In a study which followed up patients with repeated low level viraemia, levels > 200 copies/mL were associated with an increased chance of future virological failure [21]. However, this was not the case for patients with “blips” or levels <200 copies/mL. The risk of failure in the next 18 months was the same as that of patients with consistently undetectable viral loads during clinical follow up. Thus levels below this cut off are unlikely to be clinically significant and require no clinical action. They may well reflect inadvertent detection of cell associated viral nucleic acids during testing.

### Limitations

Due to the design of the study, where routine diagnostic samples were used, direct assessment of viral decay using the same set of patient samples in the different experiments was not possible. Also only laboratory conditions were tested. Further field studies are required to confirm our findings. Despite this, we believe the relatively large sample size in this study contributes substantially to the existing data around VL stability. Samples were selected on the basis of having sufficient plasma for a second test and this could be a source of bias, however, this is likely to be random and should not result in significant over or under estimation of VL decay. As we selected more samples with detectable VL, in the interest of having sufficient power to analyse VL decay, the impact of the VL delay could be overestimated. Finally, because we performed a second VL with limited plasma in the primary tube, the study design could have increased the chances of detecting cell associated viral nucleic acids in the second test.

## Conclusions

Despite the limitations that could increase the variance in VL between test 1 and 2, viral RNA in samples stored in primary tubes is remarkably stable under sub-optimal storage conditions. Samples stored for up to a week reliably differentiated between ART-suppressed and failing patients in 98.83% of instances. The impact of the delay on EDTA and PPT tubes were largely equivalent, although there was slightly more variability with EDTA samples. Re-centrifugation prior to repeat testing is very important to minimise inadvertent detection of cell associated viral nucleic acids which falsely elevates the viral load in some samples. Low RNA levels (<2 log copies/mL) in sub-optimally stored samples should be interpreted with caution as this could be due to cell-associated viral nucleic acids rather than true treatment failure.

## Supporting information

S1 Fig (PPTX)

S1 Table (PPTX)

S2 Table (PPTX)

## References

[1] NHLS Annual report 2017/18 [Internet]. 2018. p. 71–5. Available from: http://intranet.nhls.ac.za/assets/files/policy/NHLS_AR_2018.pdf

[2] Abbott RealTime HIV1 kit insert 51-602100/R10 [Internet]. 2014. Available from: https://www.who.int/diagnostics_laboratory/evaluations/pq-list/hiv-vrl/160530_0145_027_00_final_public_report_v2.pdf

[3] Roche CAP/CTM HIV-1 v2.0 EXPT-IVD [Internet]. 2018. Available from: https://pim-eservices.roche.com/eLD_SF/za/en/Documents/GetDocument?documentId=ab57160e-0bd6-e811-df87-00215a9b3428

[4] Amellal B, Murphy R, Maiga A, Brucker G, Katlama C, Calvez V, et al Stability of HIV RNA in plasma specimens stored at different temperatures. HIV Med. 2008;9(9):790–3. 10.1111/j.1468-1293.2008.00632.x 18754803

[5] Vandamme AM, Van Laethem K, Schmit JC, Van Wijngaerden E, Reynders M, Debyser Z, et al Long-term stability of human immunodeficiency virus viral load and infectivity in whole blood. Eur J Clin Invest. 1999;29(5):445–52. 10354202 10.1046/j.1365-2362.1999.00462.x

[6] Bonner K, Siemieniuk RA, Boozary A, Roberts T, Fajardo E, Cohn J. Expanding access to HIV viral load testing: A systematic review of RNA stability in EDTA tubes and PPT beyond current time and temperature thresholds. PLoS One. 2014;9(12):1–13. 10.1371/journal.pone.0113813 PMC4249975 25437009

[7] Goedhals D, Scott LE, Moretti S, Cooper MA, Opperman WJL, Rossouw I. Evaluation of the use of plasma preparation tubes for HIV viral load testing on the COBAS AmpliPrep/COBAS TaqMan HIV-1 version 2.0. J Virol Methods [Internet]. 2013;187(2):248–50. Available from: 10.1016/j.jviromet.2012.11.019 23178587

[8] Procop GW, Taege AJ, Starkey C, Tungsiripat M, Warner D, Schold JD, et al Preanalytic process linked to spuriously elevated HIV viral loads: improvement on an FDA-approved process. Diagn Microbiol Infect Dis. 2017;89(1):44–6. 10.1016/j.diagmicrobio.2016.09.003 28647065

[9] Kran AMB, Jonassen TØ, Sannes M, Jakobsen K, Lind A, Mæland A, et al Overestimation of human immunodeficiency virus type 1 load caused by the presence of cells in plasma from plasma preparation tubes. J Clin Microbiol. 2009;47(7):2170–4. 10.1128/JCM.00519-09 19420166 PMC2708492

[10] Giordano M, Kelleher T, Colonno RJ, Lazzarin A, Squires K. The effects of the Roche AMPLICOR HIV-1 MONITOR® UltraSensitive Test versions 1.0 and 1.5 viral load assays and plasma collection tube type on determination of response to antiretroviral therapy and the inappropriateness of cross-study comparisons. J Clin Virol. 2006;35(4):420–5. 16604577 10.1016/j.jcv.2005.10.011

[11] Kraft CS, Binongo JNG, Burd EM, Eaton ME, McCloskey CB, Fernandes H, et al Successful use of Plasma Preparation Tubes^TM^ (PPTs) in the COBAS®AmpliPrep/COBAS®TaqMan®HIV-1 Test. J Clin Virol [Internet]. 2013;57(1):77–9. Available from: 10.1016/j.jcv.2012.12.015 23332979 PMC3684267

[12] Douglas Wilson, Sudeshni Naidoo, Linda-Gail Bekker, Mark Cotton GM. Handbook of HIV Medicine 1st ed. Douglas Wilson, Sudeshni Naidoo, Linda-Gail Bekker, Mark Cotton GM, editor. Oxford New York: Oxford University Press, South Africa; 2002 44–46 p.

[13] Ellman TM, Alemayehu B, Abrams EJ, Arpadi S, Howard AA, El-Sadr WM. Selecting a viral load threshold for routine monitoring in resource-limited settings: Optimizing individual health and population impact: Optimizing. J Int AIDS Soc. 2017;20:16–8. 10.1002/jia2.25007 PMC5978659 29171192

[14] Neil T. Parkin. Measurement of HIV-1 Viral Load for Drug Resistance Surveillance using Dried Blood Spots: Literature Review and Modeling of Contribution of DNA and RNA. AIDS Rev [Internet]. 2014;16(3). Available from: http://www.aidsreviews.com/resumen.php?id=1272&indice=2014163&u=unp 25221990

[15] Lee TH Stromberg RR Heitman JW Sawer L Hanson CV Busch M. Distribution of HIV type 1 (HIV-1) in blood components: detection and significance of high levels of HIV-1 associated with platelets. Transfusion. 1998;38(6):580–8. 9661692 10.1046/j.1537-2995.1998.38698326338.x

[16] EC Sabino N Gaburo JP Leiti M Viecili LR. Platelets May Affect Detection and Quantitation of HIV RNA in Plasma Samples. J Acquir Immune Defic Syndr. 2004;37(3):1431–3. 15483474 10.1097/00126334-200411010-00013

[17] Giuseppe Banfi, Gian Luca Salvagno GL. The role of ethylenediamine tetraacetic acid (EDTA) as invitro anticoagulant for diagnostic purposes. Clin Chem Lab Med. 2007;45(5):565–76. 10.1515/CCLM.2007.110 17484616

[18] Baca A, Haber RJ, Sujishi K, Frost PH, Ng VL. Artifactual Undetectable HDL-Cholesterol with the Beckman Synchron LX and Vitros 950 Assays Temporally Associated with a Paraprotein [1]. Clin Chem. 2004;50(1):255–6. 10.1373/clinchem.2003.027813 14709669

[19] Besson GJ, Lalama CM, Bosch RJ, Gandhi RT, Bedison MA, Aga E, et al HIV-1 DNA decay dynamics in blood during more than a decade of suppressive antiretroviral therapy. Clin Infect Dis. 2014;59(9):1312–21. 10.1093/cid/ciu585 25073894 PMC4200019

[20] Golob JL, Stern J, Holte S, Kitahata MM, Crane HM, Coombs RW, et al HIV DNA levels and decay in a cohort of 111 long-term virally suppressed patients. Aids [Internet]. 2018;(1):1 Available from: http://insights.ovid.com/crossref?an=00002030-900000000-97148 10.1097/QAD.0000000000001674 30005008 PMC6136948

[21] Esber A, Polyak C, Kiweewa F, Maswai J, Owuoth J, Maganga L, et al Persistent low level viremia predicts subsequent virologic failure. Is it time to change the 3rd 90? Clin Infect Dis [Internet]. 2018;(7 2018):23–7. Available from: https://academic.oup.com/cid/advance-article/doi/10.1093/cid/ciy989/5193460 10.1093/cid/ciy989 30462188

