# Supplementary material for: Reliability of plasma HIV viral load testing beyond 24 hours: Insights gained from a study in a routine diagnostic laboratory
Source: PLoS One. 2019 Jul 3;14(7):e0219381. doi: 10.1371/journal.pone.0219381 (PMC6609026; doi:10.1371/journal.pone.0219381)
Supplement: S1 Fig — (PPTX) [file pone.0219381.s001.pptx]

## Slide 1
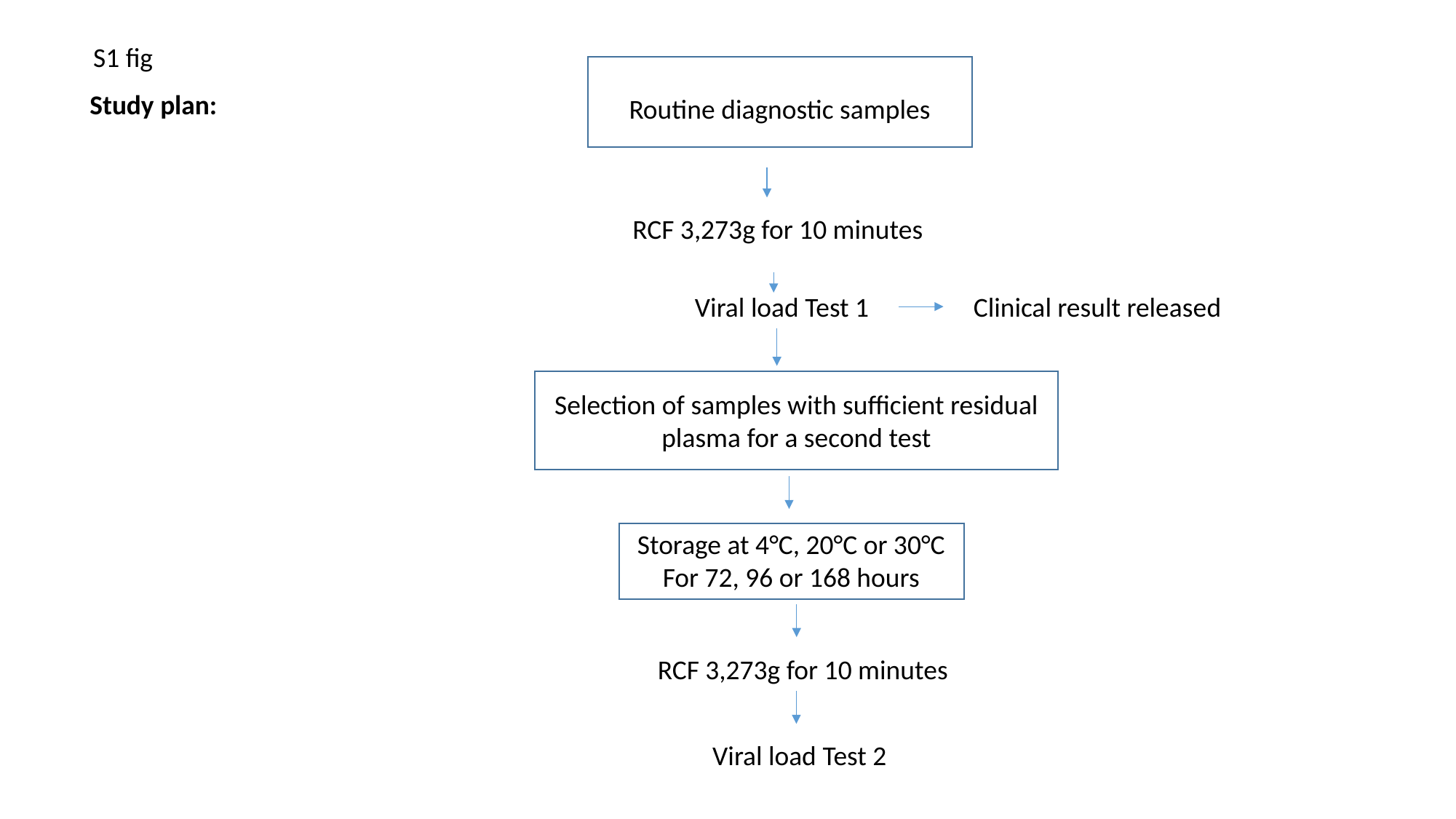

S1 fig
Routine diagnostic samples
RCF 3,273g for 10 minutes
Viral load Test 1
Selection of samples with sufficient residual plasma for a second test
Storage at 4°C, 20°C or 30°C
For 72, 96 or 168 hours
RCF 3,273g for 10 minutes
Viral load Test 2
Study plan:
Clinical result released
