# Supplementary material for: Reliability of plasma HIV viral load testing beyond 24 hours: Insights gained from a study in a routine diagnostic laboratory
Source: PLoS One. 2019 Jul 3;14(7):e0219381. doi: 10.1371/journal.pone.0219381 (PMC6609026; doi:10.1371/journal.pone.0219381)
Supplement: S1 Table — (PPTX) [file pone.0219381.s002.pptx]

## Slide 1
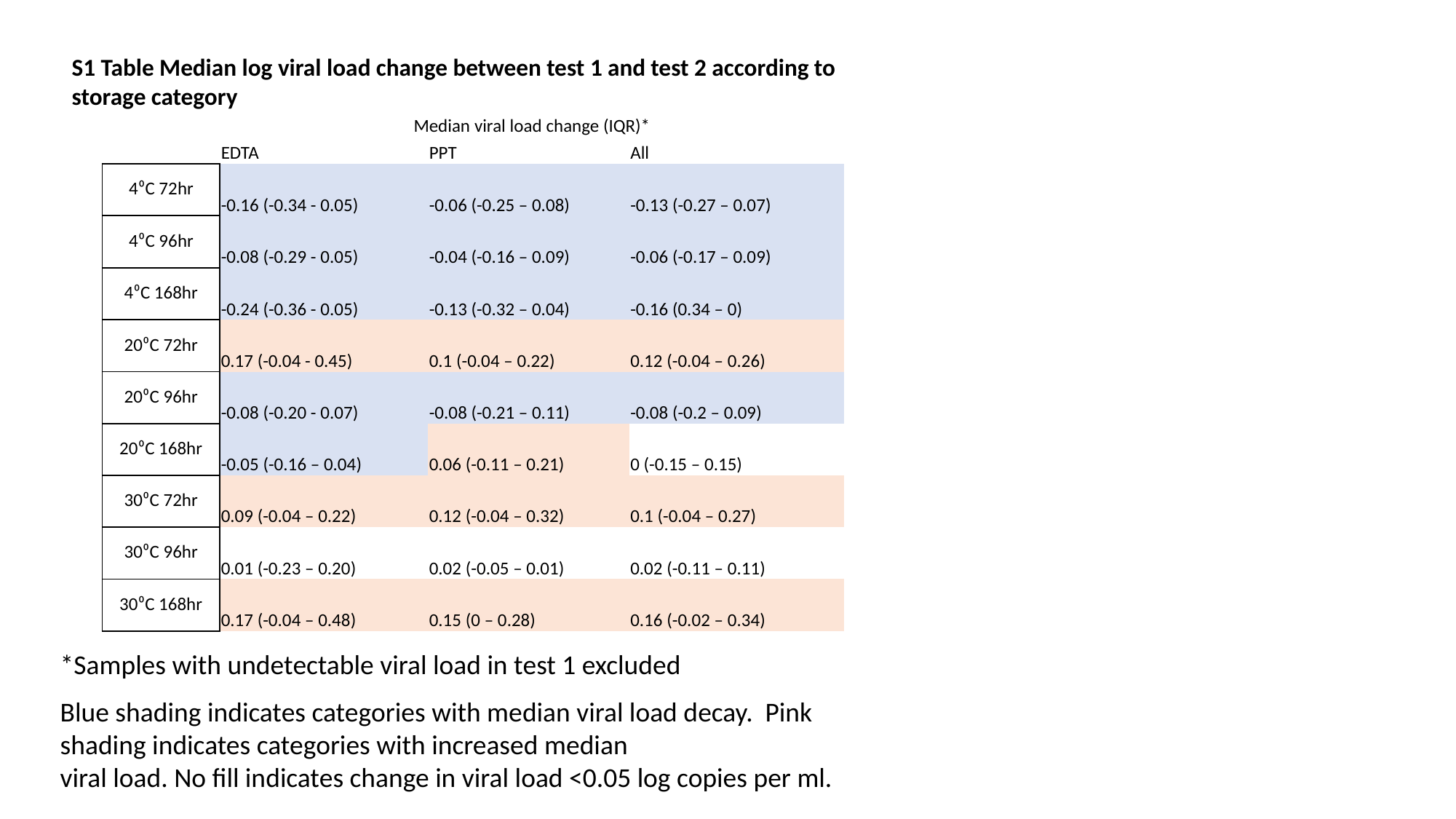

S1 Table Median log viral load change between test 1 and test 2 according to
storage category
| | | | | | |
| --- | --- | --- | --- | --- | --- |
| | Median viral load change (IQR)\* | | | | |
| | EDTA | | PPT | All | |
| 4⁰C 72hr | -0.16 (-0.34 - 0.05) | | -0.06 (-0.25 – 0.08) | -0.13 (-0.27 – 0.07) | |
| 4⁰C 96hr | -0.08 (-0.29 - 0.05) | | -0.04 (-0.16 – 0.09) | -0.06 (-0.17 – 0.09) | |
| 4⁰C 168hr | -0.24 (-0.36 - 0.05) | | -0.13 (-0.32 – 0.04) | -0.16 (0.34 – 0) | |
| 20⁰C 72hr | 0.17 (-0.04 - 0.45) | | 0.1 (-0.04 – 0.22) | 0.12 (-0.04 – 0.26) | |
| 20⁰C 96hr | -0.08 (-0.20 - 0.07) | | -0.08 (-0.21 – 0.11) | -0.08 (-0.2 – 0.09) | |
| 20⁰C 168hr | -0.05 (-0.16 – 0.04) | | 0.06 (-0.11 – 0.21) | 0 (-0.15 – 0.15) | |
| 30⁰C 72hr | 0.09 (-0.04 – 0.22) | | 0.12 (-0.04 – 0.32) | 0.1 (-0.04 – 0.27) | |
| 30⁰C 96hr | 0.01 (-0.23 – 0.20) | | 0.02 (-0.05 – 0.01) | 0.02 (-0.11 – 0.11) | |
| 30⁰C 168hr | 0.17 (-0.04 – 0.48) | | 0.15 (0 – 0.28) | 0.16 (-0.02 – 0.34) | |
*Samples with undetectable viral load in test 1 excluded
Blue shading indicates categories with median viral load decay. Pink
shading indicates categories with increased median
viral load. No fill indicates change in viral load <0.05 log copies per ml.
