# Supplementary material for: Reliability of plasma HIV viral load testing beyond 24 hours: Insights gained from a study in a routine diagnostic laboratory
Source: PLoS One. 2019 Jul 3;14(7):e0219381. doi: 10.1371/journal.pone.0219381 (PMC6609026; doi:10.1371/journal.pone.0219381)
Supplement: S2 Table — (PPTX) [file pone.0219381.s003.pptx]

## Slide 1
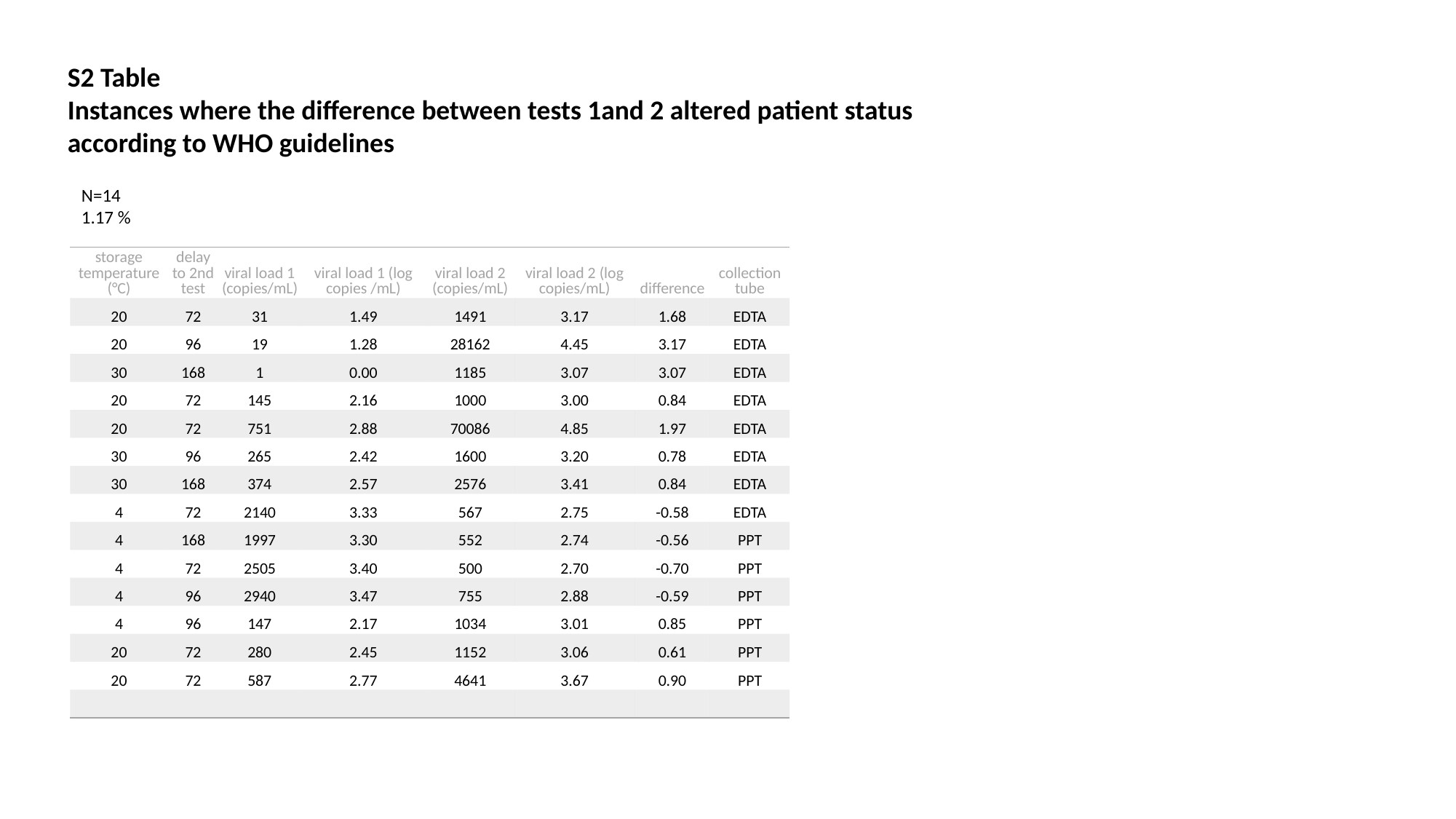

S2 Table
Instances where the difference between tests 1and 2 altered patient status
according to WHO guidelines
N=14
1.17 %
| storage temperature (°C) | delay to 2nd test | viral load 1 (copies/mL) | viral load 1 (log copies /mL) | viral load 2 (copies/mL) | viral load 2 (log copies/mL) | difference | collection tube |
| --- | --- | --- | --- | --- | --- | --- | --- |
| 20 | 72 | 31 | 1.49 | 1491 | 3.17 | 1.68 | EDTA |
| 20 | 96 | 19 | 1.28 | 28162 | 4.45 | 3.17 | EDTA |
| 30 | 168 | 1 | 0.00 | 1185 | 3.07 | 3.07 | EDTA |
| 20 | 72 | 145 | 2.16 | 1000 | 3.00 | 0.84 | EDTA |
| 20 | 72 | 751 | 2.88 | 70086 | 4.85 | 1.97 | EDTA |
| 30 | 96 | 265 | 2.42 | 1600 | 3.20 | 0.78 | EDTA |
| 30 | 168 | 374 | 2.57 | 2576 | 3.41 | 0.84 | EDTA |
| 4 | 72 | 2140 | 3.33 | 567 | 2.75 | -0.58 | EDTA |
| 4 | 168 | 1997 | 3.30 | 552 | 2.74 | -0.56 | PPT |
| 4 | 72 | 2505 | 3.40 | 500 | 2.70 | -0.70 | PPT |
| 4 | 96 | 2940 | 3.47 | 755 | 2.88 | -0.59 | PPT |
| 4 | 96 | 147 | 2.17 | 1034 | 3.01 | 0.85 | PPT |
| 20 | 72 | 280 | 2.45 | 1152 | 3.06 | 0.61 | PPT |
| 20 | 72 | 587 | 2.77 | 4641 | 3.67 | 0.90 | PPT |
| | | | | | | | |
